# Structures Showing Negative Correlations of Signal Intensity with Postnatal Age on T_1_-weighted Imaging of the Brain of Newborns and Infants

**DOI:** 10.2463/mrms.mp.2015-0168

**Published:** 2017-02-16

**Authors:** Saeka Hori, Toshiaki Taoka, Tomoko Ochi, Toshiteru Miyasaka, Masahiko Sakamoto, Katsutoshi Takayama, Takeshi Wada, Kaoru Myochin, Yukihiro Takahashi, Kimihiko Kichikawa

**Affiliations:** 1 Department of Radiology, Nara Medical University, 840 Shijo-cho, Kashihara, Nara 634-8522, Japan; 2 Department of Radiology, Nagoya University, Aichi, Japan; 3 Department of Pediatrics, Nara Medical University, Nara, Japan

**Keywords:** newborn, brain, magnetic resonance imaging, T_1_-weighted images, development

## Abstract

**Purpose:**

Although the neonatal and infantile brain typically shows sequential T_1_ shortening according to gestational age as a result of myelination, several structures do not follow this rule. We evaluated the relationship between the signal intensity of various structures in the neonatal and infantile brain on T_1_-weighted imaging (T_1_WI) and either postnatal or gestational age.

**Materials and Methods:**

We examined magnetic resonance images from 120 newborns and infants without any abnormalities in the central nervous system. Written informed consent was obtained from all parents and the institutional review board approved the study. Gestational age at examination ranged from 35 weeks, 3 days to 46 weeks, 6 days, and postnatal age ranged from 7 days to 127 days. Signal intensity on T_1_WI was evaluated on a scale from Grade 1 (indistinguishable from surrounding structures) to Grade 4 (higher than cortex and close to fat). We evaluated relationships between the T_1_ signal grades of various structures in the neonatal brain and postnatal or gestational age using Spearman’s correlation analysis.

**Results:**

Significant positive correlations were identified between T_1_ signal grade and gestational age in the pyramidal tract (*P* < 0.001). Conversely, significant negative correlations were evident between T_1_ signal grade and postnatal age (*P* < 0.001), in structures including the stria medullaris thalami, fornix cerebellar vermis, dentate nucleus and anterior pituitary gland.

**Conclusion:**

Significant negative correlations exist between signal intensity on T_1_WI and postnatal age in some structures of the neonatal and infantile brain. Some mechanisms other than myelination might play roles in the course of signal appearance.

## Introduction

Signal intensity on the T_1_-weighted image (T_1_WI) of the neonatal brain is widely recognized as developing from hypointensity to hyperintensity according to gestational age, in a process that is attributed to myelination.^1^ However, recent reports have demonstrated that some structures show hyperintensity on T_1_WI soon after birth and a subsequent decrease in signal intensity according to postnatal age. These structures include the anterior lobe of the pituitary gland, subthalamic nucleus, and globus pallidus.^2–4^ This signal intensity pattern, hyperintensity to hypointensity according to postnatal age, is speculated to be due to different mechanisms from myelination. For example, the hyperintensity of the pituitary gland just after birth is reported to be due to the hyperplasia of prolactin cells.^2^ In our previous report which pointed out T_1_ hyperintensity in the subthalamic nucleus and globus pallidus just after the birth, we made speculation about the mechanism that might be due to various processes other than myelination including the rapid proliferation of oligodendroglial cells, cerebral glial reaction by stressful environment at delivery or influence from maternal thyroid hormone.^4^

Above mentioned speculation made us consider that there would be other structures in the brain which shows a similar signal transition pattern, because the mechanism we speculated might occur in anywhere in the brain. And it would be important to be aware of the distribution of those structures.

We selected following structures to evaluate the time course. As the basis of our discussions, we evaluated the structures which show typical and early signal transition by myelination called as “early myelinators” such as pyramidal tract, and “intermediate myelinator” that is the corpus callosum.^5^ The structures of limbic system were selected because limbic system are formed earlier than the structures related to neocortex, and may show different tissue development compared to the early or intermediate myelinator structure. In addition, several structures in the cerebellum were also evaluated in order to myelination related to the cerebellum. In the current study, we examined the signal intensities of the above-mentioned structures in the neonatal and infantile brain on T_1_WI and evaluated correlations between signal intensities and postnatal or gestational age.

## Materials and Methods

### Subjects

Subjects were newborns and infants who had been admitted to the neonatal intensive care unit (NICU) of our institute and showed normal development throughout two years of follow-up. We retrospectively analyzed 170 consecutive newborns admitted to the NICU for perinatal troubles who had undergone screening magnetic resonance imaging (MRI) of the brain between January 1, 2007 and August 7, 2008. Written informed consent was obtained from all parents prior to the enrollment of subjects and all study protocols were approved by the institutional review board in our institute. Reasons for MRI were as follows: low birth weight or premature birth, 140 cases; neonatal asphyxia, 11 cases; respiratory distress syndrome, 2 cases; transient tachypnea of the newborn, 6 cases; intrauterine growth restriction, 2 cases; ABO incompatibility, 2 cases; congenital diaphragmatic hernia, 2 cases; congenital esophageal atresia, 3 cases; pneumothorax, 1 case; and meningocele, 1 case. From this population, we excluded 18 cases with pathological status detected in the central nervous system: congenital cytomegalovirus infection, 1 case; hydrocephalus, 2 cases; intracranial hematoma, 1 case; subependymal hemorrhage, 1 case; periventricular leukomalacia, 2 cases; chromosomal abnormality, 3 cases; Langerhans cell histiocytosis, 1 case; myotonic dystrophy, 2 case; hyperinsulinemia, 1 case; the epilepsy of unknown cause, 3 case; and congenital ichthyosis, 1 case. We also excluded 11 cases with developmental abnormalities based on evaluation with the Enjoji Infantile Developmental Test.^6^ We also excluded 21 cases that were lost to follow-up two years after birth. In total, we excluded 50 patients over the two years of follow-up. Ultimately, 120 newborns and infants were identified as showing normal development with normal findings on both MRI and developmental testing throughout the two years of follow-up.

We defined the terms concerning the chronological age of the newborn in the current study as follows. Gestational age to refer to the age of the newborn at birth based on the number of weeks gestation, i.e. from the first day of the last menstrual period to the day of delivery, and postnatal age to refer to the period from birth to the day of the MRI examination. In the current study, the gestational age ranged from 248 to 328 days (mean: 272.0 days, median: 268 days), and the postnatal age ranged from 8 to 119 days (mean: 33.1 days, median: 26 days).

### Imaging and data analysis

MRI was performed using a 1.5T clinical MR unit (Magnetom Sonata; Siemens AG, Erlangen, Germany) with a standard 8-channel head coil. All examinations included axial sections perpendicular to the brainstem with conventional spin-echo T_1_-weighted (repetition time, 500 ms; echo time, 12 ms; averaging, 1; acquisition time, 3 min 26 sec). Section thickness was 6 mm with a 2 mm gap. Images were obtained using a 256 × 256 displayed matrix and a 230-mm field of view. Images were acquired during natural sleep with some foam cushions to provide some restraint.

**Fig 1.**
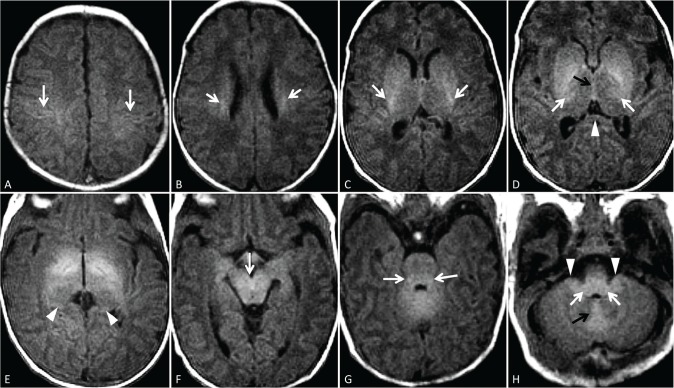
Structures of interest. (**A**, **B**) Images at the level of the convexity show the precentral gyrus (white arrows in **A**), and the pyramidal tract in the corona radiata (white arrows in **B**). (**C**, **D**, **E**) Images at the level of the basal ganglia show the posterior limb of the internal capsules (white arrows in **C**), the ventrolateral thalamic nucleus (white arrows in **D**), the stria medullaris thalami (black arrow in **D**), the corpus callosum (white arrowhead in **D**) and the fornix (white arrowheads in **E**). (**F**) Image at the level of the midbrain shows the decussation of the superior cerebellar peduncles (white arrow). (**G**) Image at the level of the midpons shows the superior cerebellar peduncles (white arrows) at the lateral aspects of the fourth ventricle. (**H**) Image at the level of the medulla shows the flocculus cerebellum (white arrowheads), inferior cerebellar peduncles (white arrows), and cerebellar vermis (black arrow).

**Fig 2.**
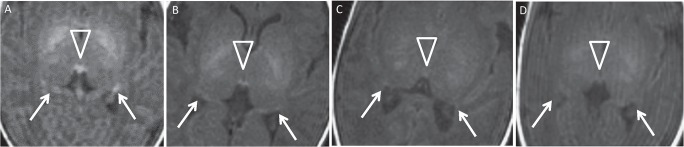
Grading of T_1_ signal intensity. Qualitative evaluation of T_1_ signal intensity in the medullaris thalami (arrowheads) and fornix (arrows). Grading of T_1_ signal intensity is as follows: Grade 4, signal intensity higher than cortex and close to fat (**A**); Grade 3, signal intensity higher than cortex (**B**); Grade 2, signal intensity between cortex and surrounding structures (**C**); Grade 1, signal intensity indistinguishable from surrouding structures (**D**).

MR images were retrospectively and independently reviewed by two radiologists (S.H., T.T). The radiologists were blinded to the ages of newborns and infants. We selected the following structures bilaterally (Fig. 1): [Early myelinator and intermediate myelinator] pyramidal tract in the precentral gyrus, corona radiata and posterior limb of the internal capsule; corpus callosum, [Limbic system] fornix; stria medullaris thalami; [Cerebellar structure] cerebellar vermis; dentate nucleus of the cerebellum; the decussation of the superior cerebellar peduncles; flocculus cerebellum; superior cerebellar peduncle; inferior cerebellar peduncle; [Other] the ventrolateral nucleus of the thalamus; and anterior pituitary gland. We examined the signal intensity on T_1_WI of each structure and qualitatively classified signal intensity on T_1_WI into 4 grades (Fig. 2): Grade 1, indistinguishable from surrounding structures; Grade 2, intensity between the cortex and surrounding structures; Grade 3, higher intensity than cortex, but close to the cortex; Grade 4, higher intensity than the cortex and close to fat. We evaluated the relationship between T_1_ signal grade and gestational or postnatal age using Spearman’s rank correlation analysis and compared correlation coefficients (*rs*) between gestational age and postnatal age in each structure. The strength of a calculated absolute *rs* value was interpreted as follows: 0.00 to 0.19, very weak to negligible; 0.20 to 0.39, weak; 0.40 to 0.69, moderate; 0.70 to 0.89, strong; 0.90 to 1.00, very strong correlation. We evaluated *rs* values as meaningful when the strength was categorized as moderate, strong or very strong.

## Results

**Fig 3.**
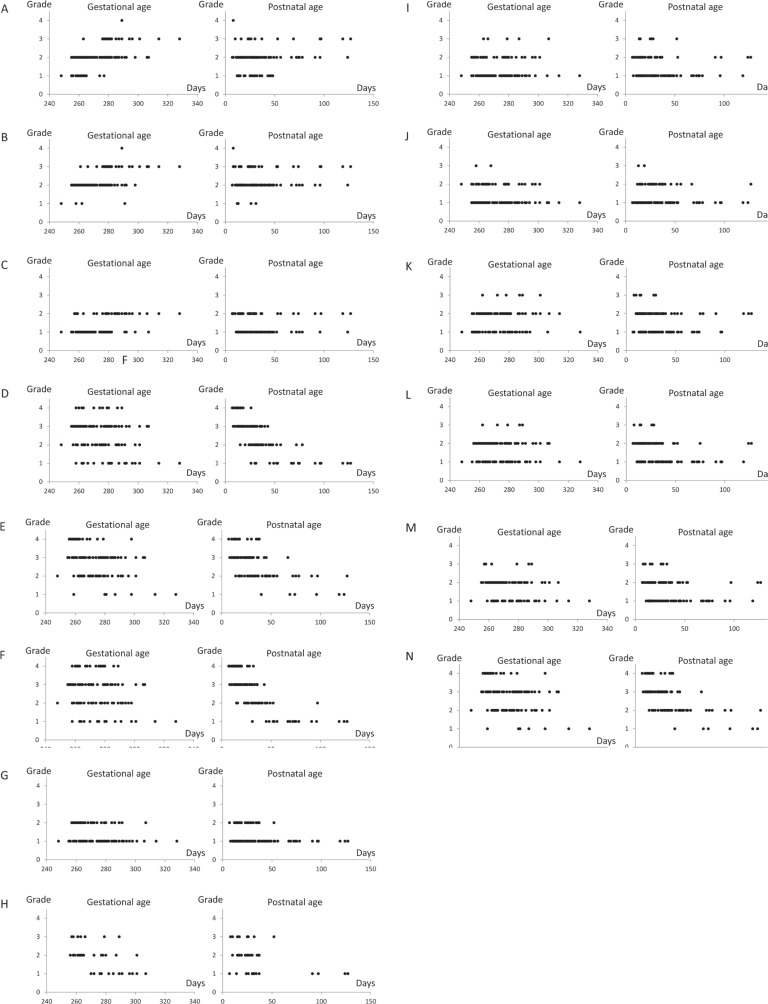
Correlation between signal grade and gestational/postnatal age. Signal grades are plotted vertically and ages are plotted horizontally. Graphs on the left side are plotted using gestational age, and graphs on the right side using postnatal ages. (**A**) Precentral gyrus, (**B**) Pyramidal tract in the corona radiata, (**C**) Posterior limb of the internal capsule, (**D**) Fornix, (**E**) Stria medullaris thalami, (**F**) Cerebellar vermis, (**G**) Dentate nucleus of the cerebellum, (**H**) Flocculus cerebellum, (**I**) Ventrolateral nucleus of the thalamus, (**J**) Corpus callosum, (**K**) Decussation of superior cerebellar peduncles. (**L**) Superior cerebellar peduncles, (**M**) Inferior cerebellar peduncles, (**N**) Anterior pituitary gland.

Figure 3 shows signal grades and gestational/postnatal ages of the structures of interest. Table 1 shows the correlation coefficient for each structure calculated between the T_1_ signal grade as mentioned above and the gestational or postnatal age. Structures showing positive correlations with gestational age were as follows. Pyramidal tract in the precentral gyrus, corona radiata and posterior limb of internal capsule showed moderate positive correlations between T_1_ signal grade and gestational age (*P* < 0.001), but no correlation between T_1_ signal grade and postnatal age.

Structures showing negative correlations with postnatal age were as follows. The fornix, stria medullaris thalami, cerebellar vermis, and anterior pituitary gland showed hyperintensity on T_1_WI soon after birth, with signal intensity decreasing according to postnatal age. In these structures, moderate to strong negative correlations were identified between T_1_ signal grade and postnatal age. In all of these structures except the fornix, weak negative correlations were noted between T_1_ signal grade and gestational age. In the fornix, no significant correlation was apparent between T_1_ signal grade and gestational age.

The dentate nucleus of the cerebellum showed a strong negative correlation with both postnatal and gestational ages. The flocculus cerebellum showed a moderate negative correlation between T_1_ grade and gestational age and no correlation with postnatal age. The ventrolateral nucleus of the thalamus, corpus callosum, the decussation of the superior cerebellar peduncles, superior cerebellar peduncle and inferior cerebellar peduncle showed either no significant correlation or only weak correlations with postnatal age and gestational age.

## Discussion

The motivation of this study is to figure out the structures which show significant negative correlations between their T_1_ signal intensities and postnatal age, not gestational age. In our previous report, T_1_ signal intensities of the subthalamic nucleus and globus pallidus showed significant negative correlations to the postnatal age, and there was a question that if there are some other structures showing a similar pattern. So, we selected following structures to evaluate the time course. As sites expected to show typical T_1_ signal changes according to gestational ages, we evaluated white matter of precentral gyrus, pyramidal tract in the corona radiata and the posterior limb of the internal capsule. These areas are called as “early mielinators” in which myelination begins before the birth and becomes histologically mature by six postnatal months.^5^ Corpus callosum was selected as an “intermediate myelinator” in which myelination begins after birth and becomes histologically mature by six postnatal months.^5^ As limbic system structure which shows early formation compared to neocortes, fornix and stria medullaris thalami are selected. Stria medullaris thalami is reported to show the onset of myelination before birth and the myelination completely in rather early period, and fornix is reported to show onset of myelination after birth and the myelination complete in rather late period.^7,8^ We also selected cerebellar structures including cerebellar vermis, dentate nucleus of the cerebellum and flocculus cerebellum, the decussation of superior cerebellar peduncles, superior cerebellar peduncles and inferior cerebellar peduncles. Ventrolateral nucleus of the thalamus was selected to represent the central gray matter to compare with the result of our previous study for subthalamic nucleus and globus pallidus.^4^ We also evaluated anterior pituitary gland, in which transient T_1_ hyperintensity has already reported and can be act as a control of the current study.^2,3^

The present study observed moderate to strong positive correlations between the T_1_ signal intensity of the pyramidal tract and gestational age, apparently corresponding to myelination. In contrast, moderate to strong negative correlations were identified between T_1_ signal intensity and postnatal age in various structures, including the fornix, medullary stria of thalamus, cerebellar vermis, dentate nucleus of the cerebellum and anterior pituitary gland. Also in our previous study on subthalamic nucleus and globus pallidus, hyperintensities on T_1_WI in these structures were observed just after birth and these hyperintensities diminished in older subjects and the disappearance of this hyperintensity was well correlated with postnatal age.^4^ One of the explanations for the negative correlation to the age which is shown in the signal intensity may be the relative signal contrast to the surrounding structure. The signal of a structure will be looking lower when the surrounding structure myelinates especially when the evaluation is undergone by comparison with surrounding structure like the current study. However, if so there should be a negative correlation to the gestational age, not postnatal age. We hypothesized that mechanisms other than myelination may play roles in these signal changes.

At birth, newborns may be under various stresses and environmental challenges, including exposure to the hyperoxic extrauterine environment.^9^ and body temperature drop immediately after birth.^10^ In order to adapt to extrauterine life, a number of changes take place in the newborn body.^11^ Maternal and fetal hormone levels also change dynamically before and after delivery. For example, a massive release of oxytocin is the trigger of parturition and crossing easily the placenta from the mother to the newborn.^12^ After birth, thyroid-stimulating hormone (TSH) and tri-iodothyronine (T3) rise sharply and concurrently in the newborn, peaking at around 2 h.^13^ The surge in TSH is believed to be due to extrauterine cooling.^14^ Glucocorticoids also increase soon after birth.^11^ We speculated that these various stresses and hormonal changes during delivery might be associated with the signal course in the current study in the following hypotheses.

In general, T_1_ shortening is caused by various factors including lipids, paramagnetic effects, the immobilization of water molecules, and relative hyperintensity. Hyperintensity on T_1_WI can be observed in various conditions in the central nervous system. The perirolandic gyrus of infants at 41–44 weeks old reportedly show hyperintensity on T_1_WI despite the relatively scant myelination, which may reflect the accelerated neuronal development associated with the rapid proliferation of oligodendroglial cells, synapses and dendrites.^15^ The periventricular hyperintensity on T_1_WI in patients with Alexander disease is known to be due to the accumulation of Rosenthal fibers, as abnormally overproduced glial fibrillary acidic protein (GFAP) in astrocytes.^16,17^ Hyperintensity on T_1_WI of the putamen in patients with hemichorea-hemiballism has been demonstrated to be caused by abundant gemistocytes, which are swollen, protein-rich astrocytes.^18^ These conditions seem to be associated with glial reactions, and can be classified as “glia-related hyperintensity”.^4^ In addition, the putamen reportedly shows transient hyperintensity on T_1_WI after transitional middle cerebral artery occlusion, which is suggested to be caused by accumulation of manganese-binding proteins.^19^

In the current study, the structures of the limbic systems including fornix and medullary stria of the thalamus as well as the dentate nucleus of the cerebellum and cerebellar vermis showed negative correlations between T_1_ signal intensity and postnatal age, which were similar tendency with subthalamic nucleus and globus pallidus in our previous study.^4^ There may be several cause for these phenomenon and following descriptions are our speculations. One of our hypotheses is that glial reaction may be associated with hyperintensity on T_1_WI. GFAP is known as a major intermediate protein of astrocytes and plays important roles in brain development. Modulators of GFAP expression reportedly include several hormones such as T3, glucocorticoids and several growth factors.^20^ Since T3 and glucocorticoids begin to increase soon after birth, as mentioned above,^11,13^ these hormones might increase GFAP levels and lead to hyperintensity on T_1_WI. Oligodendrocytes are also stimulated by several mediators, including neuregulins, and some hormones including thyroid hormone and progesterone, which might cause transient hypermyelination leading to hyperintensity on T_1_WI.^21^ These reaction might take place in the perinatal period in which various stresses and environmental challenges described above exists.

Our next hypothesis is that high concentrations of neuronal transmitters might contribute to hyperintensity on T_1_WI. For example, gamma-aminobutyric acid (GABA) is known as the primary excitatory neurotransmitter in immature neurons and switches to act as an inhibitory neurotransmitter during delivery, which has recently been reported to be triggered by massive releases of oxytocin.^12^ The subthalamic nucleus and external globus pallidus are connected to each other by both glutamatergic and GABAergic neurons. High activity might be seen in these structures in association with the switching of GABA activity, which might cause hyperintensity on T_1_WI soon after birth. Our observation on pituitary gland as a control of the current study agreed with the previous report which shows negative correlations were identified between T_1_ signal intensity and postnatal age. This transient hyperintensity in the perinatal pituitary gland is speculated to be caused by hyperplasia of prolactin cells,^2,3^ which may be another mechanism for T_1_ hyperintensity just after birth.

Several limitations to the current study must be considered. First, subjects were not completely “normal” because they had required admission to the NICU for observation following perinatal troubles. Second, this was a qualitative analysis. However, signal intensity is usually evaluated by comparison with surrounding structures at routine image interpretation in clinical practice, as shown in the current study. Third, no histological proof has been provided for these signal changes. Finally, the cause of the signal changes must be limited to speculation.

## Conclusions

In addition to previously reported subthalamic nucleus and globus pallidus, significant negative correlations between signal intensity on T_1_WI and postnatal age exist in the limbic structures including stria medullaris thalami and fornix, as well as in the cerebellar vermis, dentate nucleus and anterior pituitary gland. We speculated that some mechanism other than myelination might play a role in these signal changes. We should be aware that some structures show hyperintensity on T_1_WI soon after birth, regardless of gestational age in physiological status. We would therefore take into account not only the gestational age, but also the postnatal age for interpretation of the neonatal and infantile brain.

## Figures and Tables

**Table 1.** The correlation coefficient for each structure calculated between the T_1_ signal grade as mentioned above and the gestational/postnatal age

	Correlation coefficients with postnatal age		Correlation coefficients with gestational age	
Precentral gyrus	NS		0.53	(*P* < 0.001)
Pyramidal tract in the corona radiata	NS		0.61	(*P* < 0.001)
Posterior limb of the internal capsule	NS		0.51	(*P* < 0.001)
Fornix	−0.43	(*P* < 0.001)	NS	
Stria medullaris thalami	−0.52	(*P* < 0.001)	−0.24	(*P* < 0.01)
Cerebellar vermis	−0.47	(*P* < 0.001)	−0.28	(*P* < 0.01)
Dentate nucleus	−0.82	(*P* < 0.001)	−0.8	(*P* < 0.001)
Flocculus cerebellum	NS		−0.54	(*P* < 0.001)
Ventrolateral nucleus of the thalamus	NS		NS	
Corpus callosum	NS		NS	
Decussation of superior cerebellar peduncles	NS		NS	
Superior cerebellar peduncle	−0.35	(*P* < 0.001)	NS	
Inferior cerebellar peduncle	−0.27		−0.24	(*P* < 0.01)
Anterior pituitary gland	−0.64	(*P* < 0.001)	−0.33	(*P* < 0.001)
